# Supplementary figures and images for: Evidence of reassortment of avian influenza A (H2) viruses in Brazilian shorebirds
Source: PLoS One. 2024 May 13;19(5):e0300862. doi: 10.1371/journal.pone.0300862 (PMC11090296; doi:10.1371/journal.pone.0300862)

PB2

Tree scale: 0.1

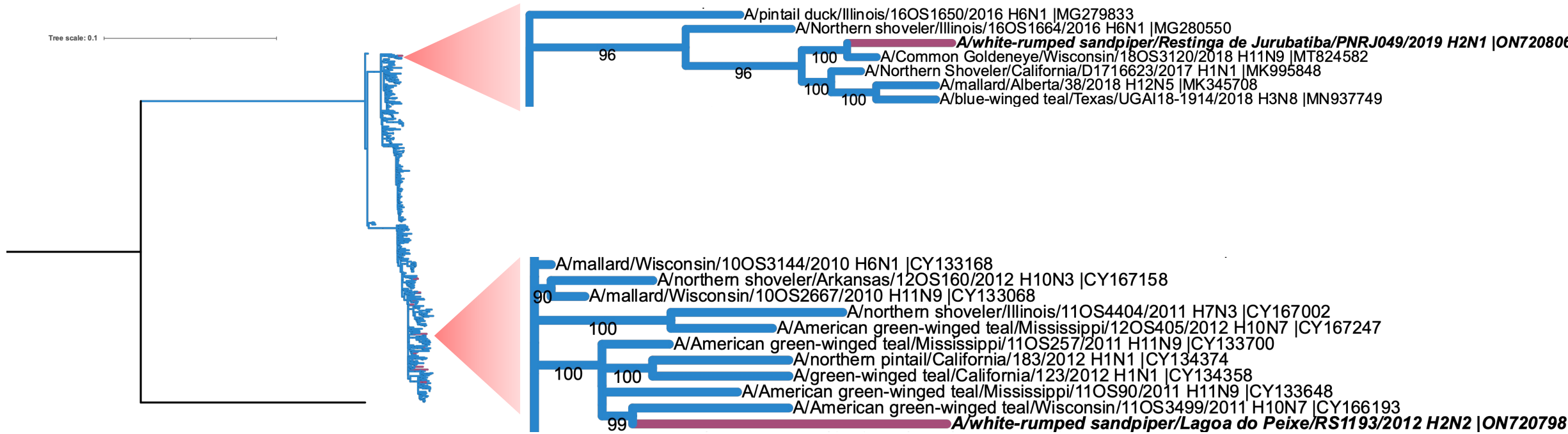

North America

South America

Supplement: S1 Fig — Brazilian samples in this study are written in a bold font. North America branches are colored blue. The GTR+F+I substitution model was used as selected by IQ-TREE Model Finder, in a 2280 nt length alignment. The scale bar represents the number of substitutions per site. Bootstraps values greater than 50% were obtained in the analysis of 1000 replicates and are presented at the branching points. The tree was rooted with the A/Korea/426/1968(H2N2) PB2 sequence (NC_007378) as the outgroup. (PDF) [file pone.0300862.s001.pdf]

PB1

Tree scale: 0.01

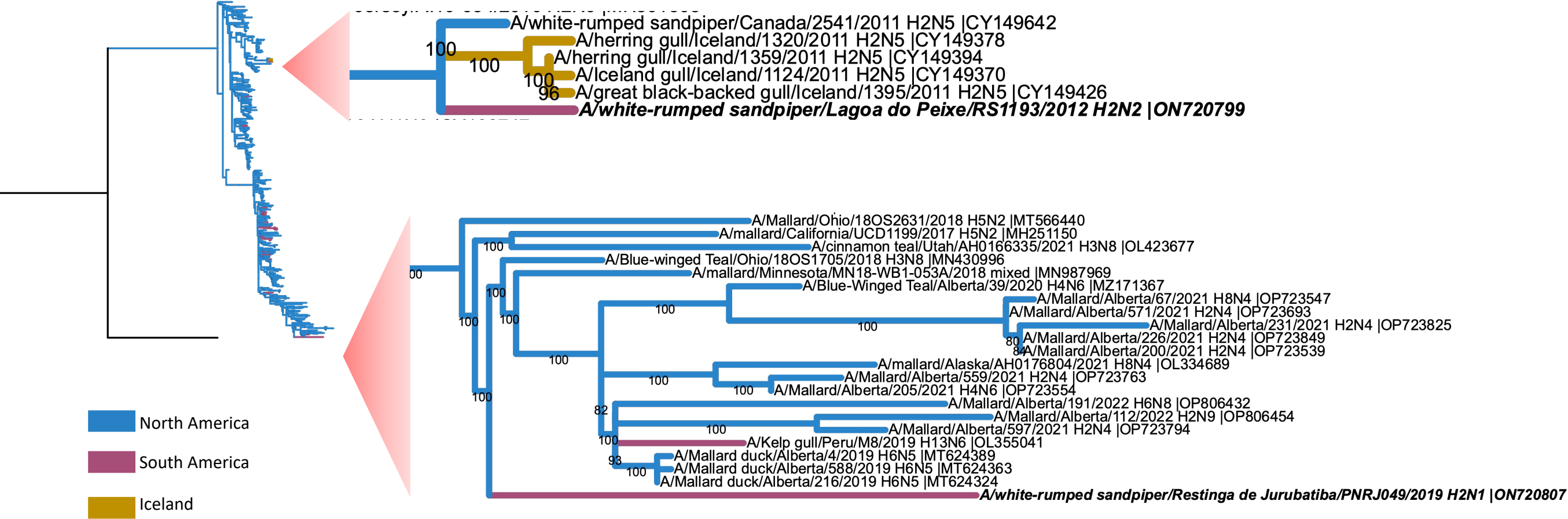

Supplement: S2 Fig — Brazilian samples in this study are written in a bold font. North America branches are colored blue, Iceland are colored gold and South America are colored purple. The GTR+F+I substitution model was used as selected by IQ-TREE Model Finder, in a 2317 nt length alignment. The scale bar represents the number of substitutions per site. Bootstraps values greater than 50% were obtained in the analysis of 1000 replicates and are presented at the branching points. The tree was rooted with the A/Korea/426/1968(H2N2) PB1 sequence (NC_007375) as the outgroup. (PDF) [file pone.0300862.s002.pdf]

NP

Tree scale: 0.1

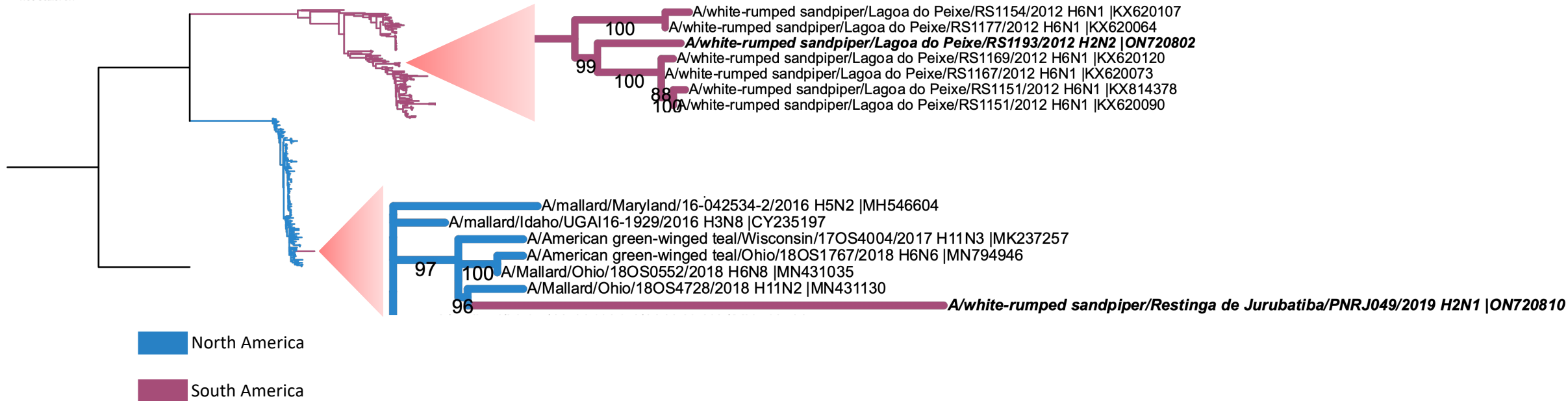

Supplement: S3 Fig — Brazilian samples in this study are written in a bold font. North America branches are colored blue and South America are colored purple. The GTR+F+I substitution model was used as selected by IQ-TREE Model Finder, in a 1497 nt length alignment. The scale bar represents the number of substitutions per site. Bootstraps values greater than 50% were obtained in the analysis of 1000 replicates and are presented at the branching points. The tree was rooted with the A/Korea/426/1968(H2N2) NP sequence (NC_007381) as the outgroup. (PDF) [file pone.0300862.s003.pdf]

M

Tree scale: 0.1

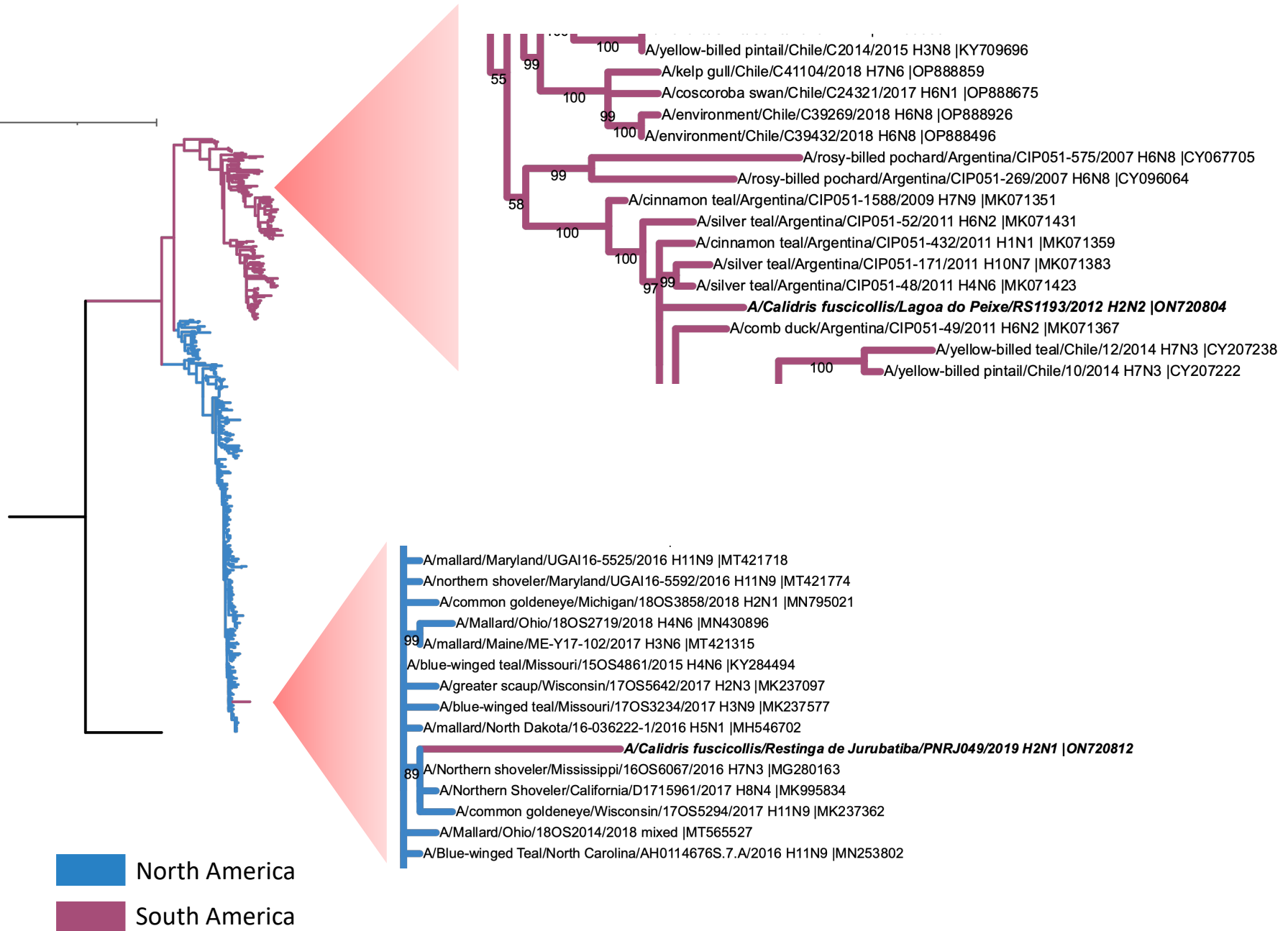

Supplement: S4 Fig — Brazilian samples in this study are written in a bold font. North America branches are colored blue and South America are colored purple. The SYM+I+I substitution model was used as selected by IQ-TREE Model Finder, in a 989 nt length alignment. The scale bar represents the number of substitutions per site. Bootstraps values greater than 50% were obtained in the analysis of 1000 replicates and are presented at the branching points. The tree was rooted with the A/Korea/426/1968(H2N2) Matrix sequence (NC_007377) as the outgroup. (PDF) [file pone.0300862.s004.pdf]

PA

Tree scale: 0.1

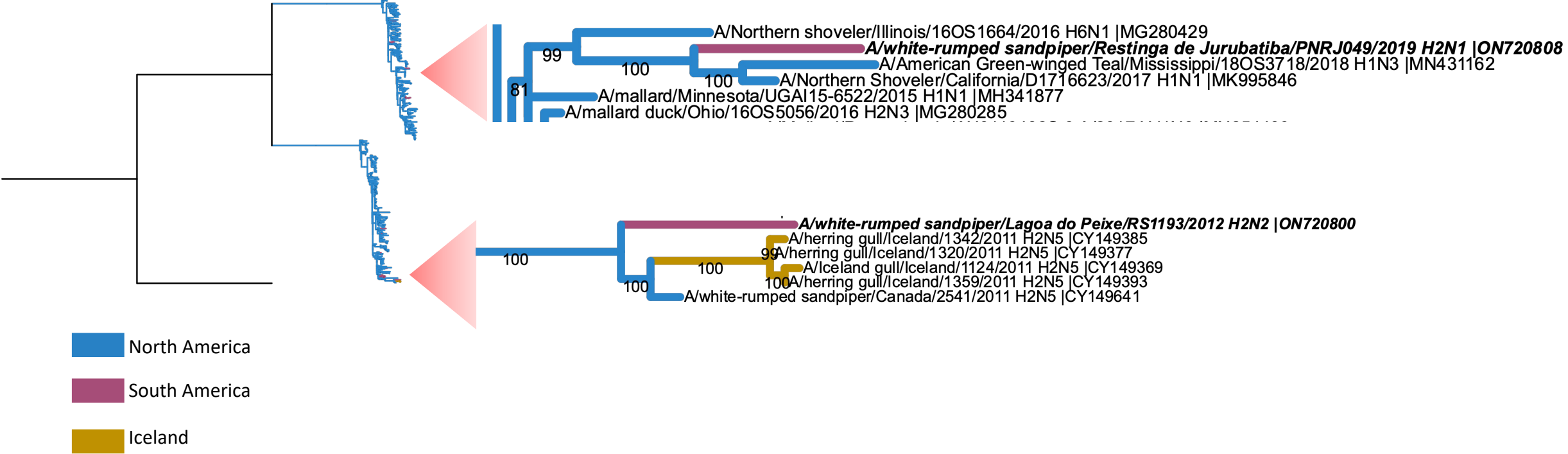

Supplement: S5 Fig — Brazilian samples in this study are written in a bold font. North America branches are colored blue, Iceland are colored gold and South America are colored purple. The GTR+F+I substitution model was used as selected by IQ-TREE Model Finder, in a 2151 nt length alignment. The scale bar represents the number of substitutions per site. Bootstraps values greater than 50% were obtained in the analysis of 1000 replicates and are presented at the branching points. The tree was rooted with the A/Korea/426/1968(H2N2) PA sequence (NC_007376) as the outgroup. (PDF) [file pone.0300862.s005.pdf]

Tree scale: 0.01

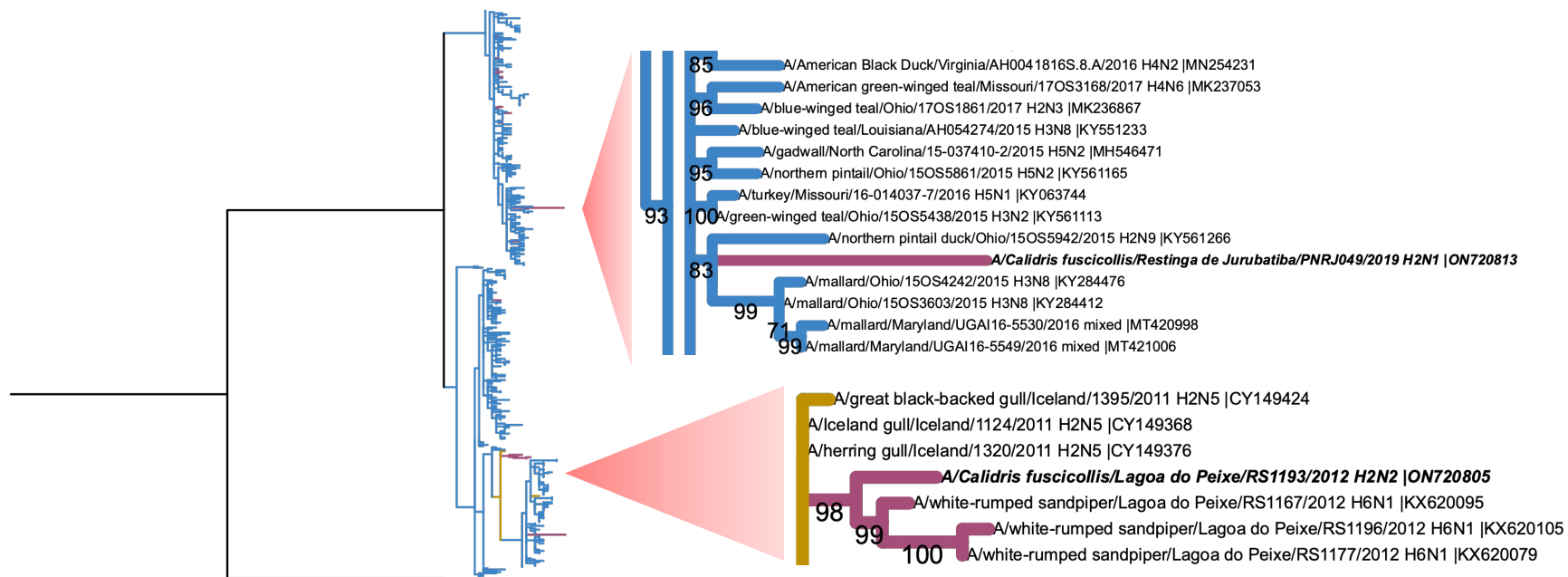

North America

South America

Iceland

Supplement: S6 Fig — Brazilian samples in this study are written in a bold font. North America branches are colored blue and South America are colored purple. The TVM+F+G substitution model was used as selected by IQ-TREE Model Finder, in an 837 nt length alignment. The scale bar represents the number of substitutions per site. Bootstraps values greater than 50% were obtained in the analysis of 1000 replicates and are presented at the branching points. The tree was rooted with the A/Korea/426/1968(H2N2) NS sequence (NC_007380) as the outgroup. (PDF) [file pone.0300862.s006.pdf]
