# Supplementary material for: Evidence of reassortment of avian influenza A (H2) viruses in Brazilian shorebirds
Source: PLoS One. 2024 May 13;19(5):e0300862. doi: 10.1371/journal.pone.0300862 (PMC11090296; doi:10.1371/journal.pone.0300862)
Supplement: Attachment — Submitted filename: Response to Reviwers H2N2 - Erick-JA revised.pdf [file pone.0300862.s007.pdf]

To the Academic Editor

Dear James Lee Crainey, Ph.D,

**Editor's report:**

PONE-D-23-19373: Evidence of reassortment of A (H2) avian influenza viruses in shorebirds in Brazil

PLOS ONE

Thank you for considering our work for potential publication in PLOS ONE. I appreciate the comments of the reviewers about our study in Brazil. We agree with all of the suggestions and believe they result in a better understanding of the work. We have complied with the suggestions, as shown in the attached, new manuscript version.

We look forward to your response,

Sincerely yours,

Jansen de Araujo, Ph.D

Institute of Biomedical Science

University of São Paulo State, Brazil

Prof. Lineu Prestes Avenue, 1374, São Paulo.

E-mail: jansentequila@usp.br

**Reviewer #1:**

The authors report the phylogenetic results of two H2 viruses found in waders in South America. I find the framing of this study strange. H2 is a common LPAI subtype in wild birds, and so is framing LPAI viruses from shorebirds as a human pandemic risk really logical? Shouldn't this be framed in the context of avian influenza and LPAI in general?

**Answer:** We totally agree that H2N2 is a LPAI found in wild birds. As we know the avian H2 virus is different genetically from the pandemic H2. However, the human H2N2 subtype disappeared after 1968, leaving no trace in the human population. Furthermore, unlike other places in the world, data on the existence of H2N2 subtypes circulating in wild birds in South America are practically non-existent. It is known that the LPAI virus is a precursor of the HPAI virus. In Brazil, the agribusiness system has increasingly advanced in green areas, and human contact has become increasingly close and frequent. The text was reformulated to improve understanding.

We included new sentence in discussion "…There are still many gaps to be filled in our understanding of viral gene flow between the two hemispheres, but demonstrates the importance of the Atlantic route as a corridor for the movement of AIVs between North America, South America and even Europe. This work came across with the scarcity of information on AIVs in South America emphasizing the necessity for additional studies on AIVs at the regional level, with a specific focus on identifying the endemic subtypes present in wild birds within the region…"

Along the same vein, the discussion is very locally focussed and doesn't draw any information from global dynamics. For example, that H2 viruses from Terns in Australia were similarly mosaic viruses seems interesting context.

**Answer:** Thank you for the excellent suggestion. We reviewed our data and included new sequences from Australia and Eurasian in the phylogenetic tree analyses (Fig 1).

Line 67. Do you really mean that these disease signs are found in waterfowl (And without a

reference)? In my experience, H2 is routinely detected and isolated from health waterfowl. I think you mean poultry?

**Answer:** We have included a new reference to this sentence “Avian A(H2) viruses in wild birds typically exhibit no pathogenicity, but they can cause symptoms like sneezing, coughing, and nasal discharge in poultry and sinusitis in waterfowl” Reference: Reneer ZB, Ross TM. H2 influenza viruses: designing vaccines against future H2 pandemics. Biochem Soc Trans 2019;47: 251–264.

Line 68. “variants” is not the correct terminology at all. You mean “subtypes”. **Answer:** We changed on text “Numerous subtypes of avian A(H2) viruses, including H2N1, H2N3, H2N5, H2N7, H2N8, and H2N9, have been reported in various animal hosts”

Line 78. This general phylogenetic division is found in most AIV subtypes, and this should be mentioned. I would say something like: Like most AIV subtypes, H2 is phylogenetic divided into two broad clades, based on geographic circulation. Unlike most AIV subtypes, however, North American sequences can be found in both the classic North America and Eurasian clades as a result of a transhemispheric transmission event and subsequent proliferation.”

**Answer:** Thanks for this point. As a suggestion, we added this sentence in our text “Like most AIV subtypes, H2 is phylogenetically divided into two broad clades, based on geographic circulation. Unlike most AIV subtypes, however, North American sequences can be found in both classic North America and Eurasian clades as a result of a trans-hemispheric transmission event and subsequent proliferation”

Line 80-81 is too vague.. what do you mean “by documented”. More detail. **Answer:** We removed this sentence.

Missing entirely is the role of shorebirds. Missing entirely is the interesting history of geographic mosasism, very much limited to gulls, terns and shorebirds. This is more relevant for this paper, than H2 human pandemic risk.

**Answer:** Thanks for the suggestion. We added a new sentence about these wild birds to the text "Some Nearctic migratory species, breeds in the Canadian Arctic from May to June and migrates to Argentine and Chilean Patagonia between January and April [14]. During its southbound migration, birds enter Brazil via the northeast coast or the mouth of the Amazon River, possibly using areas in the Pantanal Biome[15]. They arrive on the southern coast of Brazil between November and January where keep together with several species such as gulls, terns, and shorebirds with direct contact, refuel, and then continue south to Patagonia. Their extensive migratory routes and susceptibility to viruses emphasize their role as transcontinental vectors of avian viruses."

Line 104. Do you mean combined cloacal oral swabs, OR either cloacal or oral swabs were collected?

**Answer:** We corrected the sentence "…Combined cloacal/tracheal swabs samples were collected from wild birds (mainly from *Charadriiformes*) including long and intermediate migratory waterfowl and local resident…"

Line 114. Which VTM formulations?

**Answer:** We added new sentence to VTM formulations "The swabs were placed in vials containing VTM transport medium (VTM: PBS + 10% glycerol + Antibiotics/fungizone) and immediately placed in liquid nitrogen following collection".

Line 152-157. More detail about which tools and algorithms are involved here would be useful.

**Answer:** Thank you for the suggestion. More details were added to the text: **"**The Varsmetagen virome pipeline (https://varsomics.com/varsmetagen/) performed the following steps: raw sequence metrics and filtering of low quality reads (sequences with length lower than 50 bp and Phrep score lower than 20); host decontamination by mapping reads to a close host genome (Gallus gallus assembly: GCF_016699485.2) using BWA with default parameters; first round of pathogen identification using Kraken2 [20] with a custom database including Virus, Bacteria

and Human on NCBI database and complete viral genomes available in Genbank with a custom 33-kmer length; short reads assembly with Spades (version 3.13) [21]; second round of identification with contigs; search for distant homologous sequences using Hidden Markov Models (HMM) of viral proteins implemented in the eegNOG database [22] and finding confirmation through mapping and coverage metrics."

Lin e164. Why did you use the GTR+G+I for all segments. This is not in line with the best fit models for all segments, and nucleotide sub models have an impact on phylogenetic structure. Just choosing the most complex isn't necessarily the best one.

**Answer:** Thanks for suggestion. We re-run IQ-Tree with the "ModelFinder" parameter that search and uses the best model for each dataset. This information was corrected in the text. "Maximum Likelihood analysis was conducted using IQ-TREE [24] for all 8 AIV segments of each virus. The substitution model was chosen with the Model Finder parameter for each dataset." Each substitution model selected by IQ-TREE Model Finder has been presented in a new version in each figure description.

Line 167-169 is repitition of line 164.

**Answer:** The sentence was reformulated and combined in new version. "Maximum Likelihood analysis was conducted using IQ-TREE [24] for all 8 AIV segments of each virus. The substitution model was chosen with the Model Finder parameter for each dataset. Bootstrap was set to 1000 for statistical significance. Tree editing was performed with iTOL [25,26]. The tree was drawn to scale, with branch lengths measured in the number of substitutions per site. Codon positions included were 1st+2nd+3rd+Noncoding. Alignment was checked with Geneious Prime software, and all positions containing gaps and missing data were eliminated."

Lines 169-175 are not required.

**Answer:** The sentence was removed as suggested "Initial tree(s) for the heuristic search were obtained automatically by applying Neighbor-Join and BioNJ algorithms to a matrix of pairwise

distances estimated using the Maximum Composite Likelihood (MCL) approach and then selecting the topology with a superior log-likelihood value. A discrete Gamma distribution was used to model evolutionary rate differences among sites. The tree was drawn to scale, with branch lengths measured in the number of substitutions per site”.

Line 177. How did you remove gaps? Manually? Did you maybe delete a base due to poor quality gappy sequences?
**Answer:** The sequence alignment was double-checked with Geneious program. This sentenced was added: “Alignment was checked with Geneious Prime software, and all positions containing gaps and missing data were eliminated.” No base was deleted.

Line 185-188. More detail. How many samples from which species? Did you target the same populations repeatedly? How does prevalence change with time or species?
**Answer:** A total of 1212 birds were sampled at Lagoa do Peixe National Park (PNLP), belonging to different families including Ardeidae, Charadriidae, Haematopodidae, Recurvirostridae, Laridae, Rostratulidae, Tyrannidae, Furnariidae and Scolopacidae. The predominant family was Scolopacidae, with the majority being *C. fuscicolis*, accounting for 370 samples (30%). We aimed to capture distinct populations, and no animals were resampled throughout the entire collection period. In this study, we identified only one *C. fuscicollis* carrying the H2N2 subtype, representing a prevalence of 0.3% (1/370) in extreme South of Brazil. At the second site, Restinga de Jurubatiba National Park (RJNP), sampling was conducted in 2019, resulting in a total of 118 samples representing families Ardeidae, Charadriidae, Tyrannidae, Rostratulidae, Caprimulgidae, Anatidae, Motacillidae, Jacanidaea and Scolopacidae with the majority also belonging to the same species, forty-eight *C. fuscicollis* (representing 40%). All samples tested negative for avian influenza viruses, except for a single case of H2N1. Data on AIV prevalence in Brazil are still limited. So, only two wild birds (from the same species) were detected carrying the H2 subtype in South America. This unprecedented characterization is what we are presenting in the current study.

A new sentence was reformulated on text to clarify “A total 1212 oropharyngeal/cloacal samples were collected from wild birds at Lagoa do Peixe National Park (PNLP) during 2012,

belonging to different families including Ardeidae, Charadriidae, Haematopodidae, Recurvirostridae, Laridae, Rostratulidae, Tyrannidae, Furnariidae and Scolopacidae. The predominant family was Scolopacidae, with the majority being *C. fuscicolis*, accounting for 370 samples (30%). We aimed to capture distinct populations, and no animals were resampled throughout the entire collection period. In this study, we identified only one *C. fuscicollis* carrying the H2N2 subtype, representing a prevalence of 0.3% (1/370) in extreme South of Brazil. At the second site, Restinga de Jurubatiba National Park (RJNP), sampling was conducted in 2019, resulting in a total of 118 samples representing families Ardeidae, Charadriidae, Tyrannidae, Rostratulidae, Caprimulgidae, Anatidae, Motacillidae, Jacanidaea and Scolopacidae with the majority also belonging to the same species, forty-eight *C. fuscicollis* (representing 40%). All samples tested negative for avian influenza viruses, except for a single case of H2N1 (Figure 1). Only two samples, one from PNLP and one from RJNP, were positive for H2 subtype, both from free-ranging white-rumped sandpipers (*C. fuscicollis*)."

Figure 1. The pacific flyway does not include Florida. Birds also don't really fly over the open from Florida all to the way to Argentina. The Atlantic flyway doesn't start in the open ocean. I think that the flyway part of this figure needs to be reconsidered. I appreciate that its conceptual.. but it could be improved substnaitaly. The text on top of the photos is not legible. Currently not an effective figure.
**Answer:** Thanks for pointing out. The figure was improved to better understand.

Line 200. Subjected is not the correct word… Rather you generated sequences from these samples.
**Answer:** The sentence was reformulated "Both samples underwent NGS sequencing, the sequence for RJNP049 was generated from the isolated virus and PNLP1193 was generated directly from the original sample."

Line 212: Pls add the GenBank Accession for the virus from Wisconsin, from Ohio, from California etc
**Answer:** The GenBank accession numbers were included.

Table 1. The influenza designations are incorrect. Why is the sample ID at the end? It should be A/host/Country/SampleID/Year(HxNx). Please fix this. Verify that it is correct in all figures and the text.
**Answer:** Sorry about this, the table was removed as suggested by review 2.

Figure 2. The small sub-tree is too small. I cant read the tip labels so this needs to be resolved. The bright green is impossible to read.

**Answer:** Thank you for the suggestion. Indeed, the bright green was a poor choice for coloring the branches. The colors were changed for better viewing.

Can you add branch colours to the big tree too?

**Answer:** Yes, the Trees were reconstructed with all the suggestions

There is major rooting issue on the mega tree that needs to be resolved.

**Answer:** Yes, the Trees were reconstructed with all the suggestions

You need to specify in the legend how you rooted the tree.

**Answer:** Yes, the rooting specifications are now mentioned in the figure description.

What sequences are in this big tree? All H2 viruses in GenBank? Or for only certain time frames?

Answer: We used all H2 with complete cds available in NCBI Influenza Virus Database, totalizing 780 complete sequences.

And "branch lengths proportional to evolutionary distance" is vague. Should this not be "scale bar represes number of substitutions per site"? Or is IQ tree doing something different?
**Answer:** Ok, thanks for the suggestion. All legends were reformulated and included "The scale bar represents the number of substitutions per site".

Figure 3/4. Please clarify which sequences are in the mega tree. Colour branches. Rooting issues here (Fig 3, Fig 4 looks ok). Please clarify rooting. Please add a scale bar.

**Answer:** Thank you for the suggestions. The branches were colored for better clarification. The rooting was solved, and the scale bar was added in the new version.

Change NA1/NA2 to N1/N2 OR NA-N1/NA-N2.

**Answer:** Sorry to be disappointed. We corrected all in figures.

The quality is low and tip labels grainy (Figure 3, but 4 is ok). Tips colorus are inconsistent.. shoudlnt all tips be coloured. The neon green is impossible to read. Where is the scale bar? **Answer:** Thank you for the suggestion, we are submitting the trees with correct branch coloring and scale bar. In the new version.

Why is the way the format of Figure 2 different from 3/4?

**Answer:** Thank you for pointing that out. We are resubmitting the trees with the format standardized as Figure 2.

Supplemental figures consistently missing scale bars. The rooting on the large trees should be carefully assessed. There are inconsistencies in branch colouring. For some (e.g. the M tree) the tip names are not legible due to being tiny… so formatting needs to be fixed here.

**Answer:** Thank you for the suggestion. We are resubmitting the trees with better coloring, tip sizes, and rooting descriptions.

**Reviewer #2:**

I have reviewed the manuscript "Evidence of reassortment of A{H2} avian influenza viruses in shorebirds in Brazil". The authors isolated and sequenced two H2 isolates and characterized them phylogenetically, documenting that the viral genomes were derived from a variety of

geographic sources via reassortment. The paper used valid viral, genomic and phylogenetic techniques but the manuscript needs some revision, primarily to edit grammar and writing issues. I have listed several suggestions below but strongly suggest editorial review by the authors, perhaps enlisting experienced assistance.

**Answer:** Thank you for your constructive criticism to help us make this manuscript better. The lead author is currently working with the journal to ensure the figures are of adequate quality and readability for publication.

I suggest editing the title to "Evidence of reassortment of avian influenza A {H2} viruses in Brazilian shorebirds". This is more in line with accepted virus nomenclature. **Answer:** The suggestion was accepted. The title was changed "Evidence of reassortment of avian influenza A {H2} viruses in Brazilian shorebirds"

Line 63, 64 probably needs a citation citing the "concern". Maybe Joseph et al. 2015. **Answer:** Thanks for pointed. We added the reference as suggested.

Line 75. Remove "countries including"

**Answer:** The "countries including" was removed.

Line 83. I am not sure how this surveillance was "prospective" particularly since samples were collected in part, in 2012.

**Answer:** The "prospective" was removed.

Line 84. Sentence needs revision as it contains 4 "ins" within 9 words. **Answer:** This sentence was reformulated, "Over the past 15 years, we have conducted influenza surveillance in wild birds at various sites in Brazil, including shorebirds at Lagoa do Peixe National Park (PNLP) and the Amazon region."

Line 92. Can delete "however"

**Answer:** Ok. We agree, and removed “however” from the sentence.

Line 100. Should maintain past tense so change to “provided”. **Answer:** The sentence was corrected.

Lines 104 and 112. Authors use cloacal/oral and then oropharyngeal/cloacal to describe sampling technique. Which is it and be consistent. Also, line 104 swabs should be singular. **Answer:** The sentence was reformulated.

Line 114. Does the composition of VTM need to be defined? **Answer:** We added new sentence to VTM formulations “The swabs were placed in vials containing VTM transport medium (VTM: PBS + 10% glycerol + Antibiotics/fungizone) and immediately placed in liquid nitrogen following collection”.

Line 133. Virus isolation “was” attempted…

**Answer:** The sentence was corrected. “…Virus isolations was attempted…”

Delete “by standard methods” as the authors then describe technique and provide citation.

**Answer:** The “by standard methods” was removed.

Line 152. Bioinformatics should be singular. Remove “result”

**Answer:** The sentence was corrected.

Line 153. Capitalize “Varsmetagen”.

**Answer:** The sentence was corrected “Varsmetagen”.

Lines 163 and 167. Do these reference the same thing? If so then this is repetitive.

**Answer:** The sentence was combined in new version. “Maximum Likelihood analysis was conducted using IQ-TREE [24] for all 8 AIV segments of each virus. The substitution model was chosen with the Model Finder parameter for each dataset. Bootstrap was set to 1000 for

statistical significance. Tree editing was performed with iTOL [25,26]. The tree was drawn to scale, with branch lengths measured in the number of substitutions per site. Codon positions included were 1st+2nd+3rd+Noncoding. Alignment was checked with Geneious Prime software, and all positions containing gaps and missing data were eliminated."

Line 170. Should be Neighbor Joining not join

**Answer:** The sentence was reformulated. "We performed a phylogenetic analysis of the obtained IAV sequences to investigate their genetic relationship to other influenza virus sequences available in GenBank. Sequence alignments were performed using MAFFT [23]. Maximum Likelihood analysis was conducted using IQ-TREE [24] for all 8 AIV segments of each virus. The substitution model was chosen with the Model Finder parameter for each dataset. Bootstrap was set to 1000 for statistical significance. Tree editing was performed with iTOL [25,26].

The tree was drawn to scale, with branch lengths measured in the number of substitutions per site. Codon positions included were 1st+2nd+3rd+Noncoding. Alignment was checked with Geneious Prime software, and all positions containing gaps and missing data were eliminated."

Line 185. Out of 1212 swab samples only two were positive? This seems low conceptually. Were other viruses detected and if so, how many and were the H2 viruses the only ones characterized?

**Answer:** A total of 1212 birds were sampled at Lagoa do Peixe National Park (PNLP), belonging to different families including Ardeidae, Charadriidae, Haematopodidae, Recurvirostridae, Laridae, Rostratulidae, Tyrannidae, Furnariidae and Scolopacidae. The predominant family was Scolopacidae, with the majority being *C. fuscicolis*, accounting for 370 samples (30%). We aimed to capture distinct populations, and no animals were resampled throughout the entire collection period. In this study, we identified only one *C. fuscicollis* carrying the H2N2 subtype, representing a prevalence of 0.3% (1/370) in extreme South of Brazil. At the second site, Restinga de Jurubatiba National Park (RJNP), sampling was conducted in 2019, resulting in a total of 118 samples representing families Ardeidae, Charadriidae, Tyrannidae, Rostratulidae, Caprimulgidae, Anatidae, Motacillidae, Jacanidaea and Scolopacidae with the majority also belonging to the same species, forty-eight *C. fuscicollis* (representing 40%). All samples tested negative for avian influenza viruses, except for a single

case of H2N1. Data on AIV prevalence in Brazil are still limited. So, only two wild birds (from the same species) were detected carrying the H2 subtype in South America. This unprecedented characterization is what we are presenting in the current study.

A new sentence was reformulated on text to clarify "A total 1212 oropharyngeal/cloacal samples were collected from wild birds at Lagoa do Peixe National Park (PNLP) during 2012, belonging to different families including Ardeidae, Charadriidae, Haematopodidae, Recurvirostridae, Laridae, Rostratulidae, Tyrannidae, Furnariidae and Scolopacidae. The predominant family was Scolopacidae, with the majority being *C. fuscicolis*, accounting for 370 samples (30%). We aimed to capture distinct populations, and no animals were resampled throughout the entire collection period. In this study, we identified only one *C. fuscicollis* carrying the H2N2 subtype, representing a prevalence of 0.3% (1/370) in extreme South of Brazil. At the second site, Restinga de Jurubatiba National Park (RJNP), sampling was conducted in 2019, resulting in a total of 118 samples representing families Ardeidae, Charadriidae, Tyrannidae, Rostratulidae, Caprimulgidae, Anatidae, Motacillidae, Jacanidaea and Scolopacidae with the majority also belonging to the same species, forty-eight *C. fuscicollis* (representing 40%). All samples tested negative for avian influenza viruses, except for a single case of H2N1 (Figure 1). Only two samples, one from PNLP and one from RJNP, were positive for H2 subtype, both from free-ranging white-rumped sandpipers (*C. fuscicollis*)."

Line 185 should read "a total OF 1212"

**Answer:** The sentence was corrected.

Line 225. Need scientific name after species.

Answer: The sentence was added reference and scientific name. "..from Northern Shovelers (*Spatula clypeata*) in California (99.04%) and Illinois (98.76%) (MK995843.1). Genbank accession numbers."

Line 253. Should read …are a reservoir…

**Answer:** The sentence was corrected.

Line 265. Need scientific name

**Answer:** The scientific name was added. "…virus from a blackish oystercatcher (*Haematopus ater*) in Chile in 2016…"

Line 273. I found estimates of 1.3 million lives lost, not millions.

**Answer:** The sentence was corrected.

Line 276. "a lot of" seems jargony. Maybe use "considerable" or some other word instead.
**Answer:** Thanks for suggestion. We changed in new version.

Line 288. Should use a citation for the Iceland work. Dusek et al. 2014.

**Answer:** The Reference was added in new version as suggested.

Line 293. Needs a word after gene. Viral gene what?

**Answer:** The sentence was corrected "…There are still many gaps to be filled in our understanding of viral gene flow between the two hemispheres…"

Table 1 can be eliminated as it was presented in the text.

**Answer:** The table was removed as suggested.

Figure legends can be revised. I suggest revising the first sentences of all figure legends to read more like "Phylogenetic analysis of the PNRJ influenza isolate HA gene" or similar. This would read better.

**Answer:** Thanks for suggestions. All legends were reformulated.

Figure 1 legend should be "are indicated"

**Answer:** Thanks for suggestions. The legend was corrected.
