# Supplementary material for: Evidence of reassortment of avian influenza A (H2) viruses in Brazilian shorebirds
Source: PLoS One. 2024 May 13;19(5):e0300862. doi: 10.1371/journal.pone.0300862 (PMC11090296; doi:10.1371/journal.pone.0300862)
Supplement: Attachment — Submitted filename: Response to Reviwers H2N2 - 3-Luc-JA-Erick.doc [file pone.0300862.s008.doc]

To the Academic Editor

Dear James Lee Crainey, Ph.D,

**Editor’s report:**

PONE-D-23-19373: Evidence of reassortment of A (H2) avian influenza viruses in shorebirds in Brazil

PLOS ONE

Thanks again for considering our work for potential publication in PLOS ONE. I appreciate the comments of reviewer 3 about our study in Brazil. We agree with all of the suggestions and believe they result in a better understanding of the work. We have complied with the suggestions, as shown in the new manuscript version.

We look forward to your response,

Sincerely yours,

_______________________

Jansen de Araujo, Ph.D

Institute of Biomedical Science

University of São Paulo State, Brazil

Prof. Lineu Prestes Avenue, 1374, São Paulo.

E-mail: jansentequila@usp.br

**Response to Reviewers**

**Reviewer #3:** I believe the manuscript is, in general, well-organized and written in good clear easy-to-follow English and I believe the data contained in the manuscript is valuable and will be considered a welcome contribution to the field. I believe the analysis used was appropriate for the study and that the key conclusions the authors have drawn are supported by the authors data and reasonable. However, many of figures are inadequately presented and some of the interpretation of the phylogenetic analysis is incorrect. Labelling of the virus sequences and referencing of viruses is also inconsistent which makes the article unnecessarily difficult to understand in places. Below I am providing detailed comments about how I think the authors can improve their manuscript and indeed resolve their phylogenetic interpretation issues so that the authors can produce a revised manuscript that I would be delighted to recommend for publication in PLoS One.

**Answer:** Thank you for your valuable feedback. We appreciate your insights and will carefully address the concerns you raised regarding the figures and interpretation of the phylogenetic analysis. We thoroughly reviewed and made the necessary improvements to ensure the accuracy and clarity of our presentation. Your input is crucial, and we are committed to delivering a revised and improved version of the manuscript.

**Reviewer #3:** Abstract

Overall, I feel there is insufficient information about what exactly has been found. For example, there is no mention of the fact genomic sequences for these viruses have been determined and no direct mention of the results obtained from the author´s phylogenetic analysis (only a mention of the conclusions drawn from this analysis). As PLoS One allows 300-word abstracts and the current abstract is only 169 words so there is plenty of space to provide this detail. I recommend, thus, that they expanded their existing abstract to include additional details.

**Answer:** We agree with the suggestions in the abstract. We expanded additional details and included new information about our findings. The abstract now has 275-word.

**Reviewer #3:** Line 37: First sentence. The word “potential is redundant and should be deleted.

**Answer:** The word “potential” was removed as suggested.

**Reviewer #3:** Introduction:

Line 84: Although I understand what the authors intend to say here, I think clarifications are needed. I suggest the sentence “Unlike most AIV subtypes, however, North American sequences can be found in both classic North America and Eurasian clades as a result of a trans-hemispheric transmission event and subsequent proliferation” should be changed to: “Unlike most AIV subtypes, however, “North American” clade sequences can be found in both classic North America and Eurasian viral genomes as a result of a trans-hemispheric transmission event and subsequent proliferation”.

**Answer:** Thanks for this point. As a suggestion, we added this sentence in our text “Unlike most AIV subtypes, however, “North American” clade sequences can be found in both classic North America and Eurasian viral genomes as a result of a trans-hemispheric transmission event and subsequent proliferation”.

**Reviewer #3:** Line 99: Breeds should be corrected to “breed” and “migrates” to “migrate”.

**Answer:** Done.

**Reviewer #3:** Line 104: The last two sentences of the penultimate paragraph of the introduction need to be reformulated for English language clarity: “They arrive on the southern coast of Brazil between November and January where keep together with several species such as gulls, terns, and shorebirds with directly contact, refuel, and then continue south to Patagonia. Their extensive migratory routes and susceptibility to 108 viruses emphasize their role as transcontinental vectors of avian viruses.”

**Answer:** The sentence was reformulated for the English language to clarify and better understand “They arrive on the southern coast of Brazil between November and January, where they congregate with various species such as gulls, terns, and shorebirds. During this time, they engage in direct contact, refuel, and subsequently continue their migration southward to Patagonia. Given their extensive migratory routes and susceptibility to viruses, these birds play a significant role as transcontinental vectors of avian viruses (see figure 1)”

**Reviewer #3:** Lines 111-115. The first three sentences of the last paragraph of the introduction need to be revised for clarity. I suggest changing it to something like this: In this study, two A(H2) viruses were isolated from wild-caught white-rumped sandpipers (Calidris fuscicollis). One was an A(H2N1) virus obtained from a bird caught in Restinga de Jurubatiba National Park (PNRJ, 113 Rio de Janeiro); the other, an A(H2N2) virus, was isolated from a bird caught in Lagoa do Peixe National Park (PNLP, Rio Grande do Sul) in the extreme south of Brazil.

**Answer:** The sentence was substituted as a suggestion in the new version “…In this study, two A(H2) viruses were isolated from wild-caught white-rumped sandpipers (*Calidris fuscicollis*). One was an A(H2N1) virus obtained from a bird caught in Restinga de Jurubatiba National Park (PNRJ, Rio de Janeiro); the other, an A(H2N2) virus, was isolated from a bird caught in Lagoa do Peixe National Park (PNLP, Rio Grande do Sul) in the extreme south of Brazil…”.

**Reviewer #3:** Fig 1. There seems to be no reference to the bird migration routes shown in figure 1. I suggest adding a “(see figure 1)” to the end of the last sentence of the penultimate paragraph and providing some description of these routes in the figure caption. Alternatively, the authors could revise the figure (removing this information) and provide more information on the collection site, if this is the only purpose the figure is to serve.

**Answer:** Thanks for this point. As a suggestion, we added “(see Figure 1)” in this last sentence, and we have described the figure caption.

**Reviewer #3:** Methods

Phylogenetic analysis section

Line 19: It appears that the authors used nucleotide sequence alignments (not inferred protein sequences) for the phylogenic analysis; however, they don´t make this explicit. They also state they used 8 AIV segments but do not provide the sequence alignments that they used for these trees. Including the sequence alignment would allow others to build on their study and so I recommend that they are all included in the supplementary material. Failing this, I think the authors should at least provide information of the length of the sequence alignment used to construct each of their phylogenetic trees.
**Answer:** We agree on this point. We added new information in the sentence “…Maximum Likelihood analysis was conducted using IQ-TREE [24] for all 8 entire nucleotide AIV segments of each virus…”. In addition, we added the length of the sequence alignment used to construct each of the phylogenetic trees on the caption of each figure.

**Reviewer #3:** Line 192: the sentence “Bootstrap was set to 1000 for statistical significance” needs to be revised for accuracy/clarity. I suggest changing to something like: “The statistical significance of phylogenetic groupings was tested with bootstrap analysis using 1000 replicates”.

**Answer:** The sentence was changed to “The statistical significance of phylogenetic groupings was tested with bootstrap analysis using 1000 replicates” as suggested.

**Reviewer #3:** Results

Sampling
Line 218: Presumably all samples were tested with the RT-PCR described in the methods section; however, I feel I would be helpful to make this explicit here in the results section.

**Answer:** Yes, we agree. The new sentence about RT-PCR screening was added “…All samples were tested for avian influenza virus RT-PCR. Two samples, one from PNLP and one from RJNP, were positive for the H2 subtype, both from free-ranging white-rumped sandpipers (*C. fuscicollis*)…”.

**Reviewer #3:** Line 219: The reference to figure 1 at this juncture does not make sense, please delete.
**Answer:** Done. Was deleted.

**Reviewer #3:** Phylogenetic analysis section

Line 223: Presently reads thus: “Phylogenetic analysis of the A(H2N1) virus showed that its internal genes clustered not with those from viruses in South America, but with those of influenza viruses isolated from North America (Fig 2 and 3)”. I think this should be revised for clarity to something like: “Phylogenetic analysis of the A(H2N1) virus recovered from the PNRJ capture bird showed that all of its internal genes clustered not with gene sequence isolated from birds sampled in South America, but with sequences of influenza viruses isolated from birds sampled in North America (Fig 2, 3, SF1-5).

**Answer:** The sentence was reformulated as suggestion “Phylogenetic analysis of the A(H2N1) virus recovered from the PNRJ capture bird showed that all of its internal genes clustered not with gene sequences isolated from birds sampled in South America, but with sequences of influenza viruses isolated from birds sampled in North America (Fig 2, 3, SF1-5).”

**Reviewer #3:** Lines 241 to 250: The information provided here is not phylogenetic analysis or helpful for interpreting the authors data. I strongly recommend that it is deleted as some of it is, in fact, miss-leading. For example, a correct interpretation of the phylogenetic analysis shown in supplementary figure 5 is that the PA gene of A(H2N1) virus from PNRJ is equally closely related to a Mississippi isolated virus as it is to the California isolated virus that they report it as being most closely related to.
**Answer:** We thank the reviewer for the recommendations, which were considered. Initially, we used two strategies to characterize these samples: (i) phylogenetic analysis; and (ii) the identity at the nucleotide level observed between the sequences considered (in percentage). Based only on identity, it is possible to observe that the PNRJ sequence presents a greater identity value (99.33%) with the California isolate (MK995846.1) in 2017 than the PA gene (MN431162.1) in 2018 (99,02%) isolated in Mississippi. However, phylogenetic analysis indicates that the PNRJ sequence is equally related to both the Mississippi isolate and the California isolate, thus providing a clearer scenario through this inference for the evolutionary pattern observed for this highlighted clade. The phylogenetic tree was indicated in the figures on the left, and on the right, the graphic representation is a zoom-in of the larger tree, which allows us to observe the phylogenetic relationship of our sequences to other sequences previously sequenced and deposited in banks of public data. In addition, we included “Identity and Phylogenetic analysis” in the title session to better understand.

**Reviewer #3:** Lines 254 to 257: The sentence “The 255 two most phylogenetically related NA genes were from influenza viruses isolated from Northern Shovelers (Spatula clypeata) in California (99.04%) and Illinois (98.76%) (MK995843.1) (Fig 3).” Does not agree with the data presented in their figure. In their figure the virus isolated from Northern Shovelers (*Spatula clypeata*) in California (99.04%) is the most closely related database sequence used in their analysis. The authors own analysis shows there are seven other database sequences just as related to theirs as the Illinois sequence is. Please reformulate your comments accordingly.

**Answer:** Thanks for this point. The sentence was corrected to clear and removed “…Illinois (98.76%)…”. The new version represent “…The most similarity related NA gene was from influenza viruses isolated from Northern Shovelers (*Spatula clypeata*) in California (99.04%) ([MK995843.1](https://www.ncbi.nlm.nih.gov/nucleotide/MK995843.1?report=genbank&log$=nucltop&blast_rank=2&RID=N50JWRYX013)) (Fig 3).…”.

All seven sequences within the clade showed a similarity below 98.76%, as found on the Illinois sequence (MG280504). However, we realized that this statement could lead to ambiguity due to the tree structure and removed it from the text.

**Reviewer #3:** Lines 258 to 261: This sentence as it is written is also miss-leading and inaccurate. I suggest it is corrected to: “Phylogenetic analysis of the A(H2N2) virus obtained from PNLP showed that its HA clustered with multiple virus isolated from with birds sampled in Iceland and North America (Fig 2).”
**Answer:** Thanks for suggestion. The sentence was reformulated in the new version “…Phylogenetic analysis of the A(H2N2) virus obtained from PNLP showed that its HA clustered with multiple viruses isolated from birds sampled in Iceland and North America (Fig 2). Based on the identity at the nucleotide level analysis, the A(H2N2) virus showed its HA most closely with that A/lesser black-backed gull/Iceland/1597/2012 (H2N7) with 98.51% identity ([CY149380.1](https://www.ncbi.nlm.nih.gov/nucleotide/CY149380.1?report=genbank&log$=nucltop&blast_rank=2&RID=N5185KPX01N))…”

**Reviewer #3:** Lines 267 to 270: the section needs to be revise for accuracy. Deleting the text: “and the PB1 (CY149642.1) 270 and PA (CY149641.1) to and AIV isolated in Canada in 2011 (99.18% 271 and 99.14%)” from the end of the sentence could resolve this issue.

**Answer:** The sentence was deleted as suggested. The new version represent “…Other gene segments of the A(H2N2) virus were most similar to viruses detected in North America; for example, the PB2 segment was most closely related to an AIV isolated in Mississippi ([CY133700.1](https://www.ncbi.nlm.nih.gov/nucleotide/CY133700.1?report=genbank&log$=nucltop&blast_rank=2&RID=N51S2BTV01N)) in 2011 (98.83% identity) (supplemental material)….”

**Reviewer #3:** Lines 271 to 274: this sentence also needs to be modified for accuracy I suggest changing it to something like: “The NS (KX620095.1) and NP (KX620073.1) segments were most similar to Brazilian viruses also collected from Lagoa do Peixe National Park in 2012.

**Answer:** The sentence was modified to “…The NS ([KX620095.1](https://www.ncbi.nlm.nih.gov/nucleotide/KX620095.1?report=genbank&log$=nucltop&blast_rank=2&RID=N51S2BTV01N)) and NP ([KX620073.1](https://www.ncbi.nlm.nih.gov/nucleotide/KX620073.1?report=genbank&log$=nucltop&blast_rank=2&RID=N51S2BTV01N)) segments were most similar to Brazilian viruses also collected from Lagoa do Peixe National Park in 2012…”

**Reviewer #3:** Discussion

Line 286: “coast are reservoir” should be changed to: “coast are a reservoir”.

**Answer:** Done.

**Reviewer #3:** Line 292: The sentence “Of note, semipalmated sandpipers also breed 293 in the southern tundra in Canada and Alaska, and winter in 294 coastal South America” seems out of place and should be deleted.

**Answer:** The sentence was removed.

**Reviewer #3:** Line 325: This sentence needs to be reformulated for clarity: “There are still many gaps to be filled in our understanding of viral gene flow between the two hemispheres, but demonstrates the importance of the Atlantic route as a corridor for the movement of AIVs between North America, South America and even Europe.” I am not entirely sure what is being said here, but perhaps the sentence could be revised to something like this: “There are still many gaps to be filled in our understanding of viral gene flow between viruses circulating in the two hemispheres, but our work contributes to understanding of the importance of the Atlantic route as a corridor for the movement of AIVs between North America, South America and even Europe.”

**Answer:** The sentence was changed as suggested.

**Reviewer #3:** Line 330: I think the closing sentence of the discussion is both redundant and difficult to understand. I think it should be reformulated or deleted.

**Answer:** The sentence was removed.

**Reviewer #3:** Figures

Most of the figures seemed to be cropped. While this way of presenting phylogenetic results is undesirable generally, in most cases I do not believe it prevents the authors from showing their key results (i.e the phylogenetic placement of their recovered virus sequences) in this paper. It is, however, a problem for figure S4 and figure S6 where the phylogenetic placement (in a bootstrap-sported clade) of the one of the two study viruses can not be seen. Please revise all the figure captions to clarify how the images have been cropped and revise figures S4 and S6 so that PNLP isolated virus sequence in S4 can be seen in a bootstrap supported clade (and all sequences of this clade can be seen). Please also revise figure S6 similarly so that the PNRJ sequence can be seen in a bootstrap supported clade (and all sequences belonging to this clade can be seen). Also please use consistent labelling of the Matrix segment. In the text and in figure S4 it appears as only “M” but in the legend it is referred to as Matrix, without the abbreviation being highlighted. It would also be helpful in the full name of the genes were provided in all the figure captions.

**Answer:** Thanks for the suggestions. All figures and captions have been revised and greater attention was paid to figures S4 and S6 as suggested by the reviewer. These two have been re-formatted and improved for better understanding. The bootstraps are included in new version.
